# Experiences and effects of HIV-related stigma among youth living with HIV/AIDS in Western Uganda: A photovoice study

**DOI:** 10.1371/journal.pone.0232359

**Published:** 2020-04-24

**Authors:** Emmanuel Kimera, Sofie Vindevogel, Didier Reynaert, Kintu Mugenyi Justice, John Rubaihayo, Jessica De Maeyer, Anne-Mie Engelen, Khamisi Musanje, Johan Bilsen

**Affiliations:** 1 Department of Public Health, Mountains of the Moon University, Fort Portal, Uganda; 2 Social Work & E-QUAL, Faculty of Education, Health, University of Applied Sciences and Arts, Gent, Belgium; 3 Department of Public Health, Mental Health and Wellbeing research group, Vrije Universiteit Brussel, Brussels, Belgium; 4 School of Psychology, Makerere University, Kampala, Uganda; George Washington University, UNITED STATES

## Abstract

HIV-related stigma has been identified as a significant stressor affecting Quality of Life of Youth Living With HIV/AIDS (YLWHA). Gaining a contextualized understanding of how this stigma is experienced by YLWHA in Western Uganda is crucial in addressing it in this group and setting. In this study, we explored the lived experiences of YLWHA with HIV-related stigma. Photovoice was used to gain insight into the lived experiences of HIV-related stigma in 11 YLWHA (15–19 years), purposively selected from a hospital-based peer support group. Group interview transcripts, notes and photographs were subjected to phenomenological hermeneutic analysis. Encounters with enacted, anticipated and internalized stigmas and their myriad sequels were prevalent in the photos and narratives of participants. Our findings were categorized and presented in 5 main themes that were identified through the analysis: being devalued, experiencing fear, experiencing injustices, feeling lonely, and lacking future perspectives. HIV-related stigmas were experienced in various socio-ecological domains but predominantly in homes and schools that ought to be supportive surroundings for youths. A multilevel approach, targeting the entire society where the root causes of stigma can be found and specific contexts like schools and homes where youth are confronted with stigma on a daily basis is proposed for a wholistic intervention.

## Introduction

Almost half of all new HIV infections globally occur in youth aged 15–24 years [1]. In countries of Sub-Saharan Africa (SSA) like Uganda, the risk of HIV infection during adolescence is high [2]. Moreover, vertical transmissions of HIV continue to occur in these countries due to low access to perinatal health care services [3]. As a result, the prevalence of HIV in Ugandan youth is on an upward trend. Furthermore, these youth have been facilitated to live longer with HIV as a chronic infection due to Antiretroviral Therapy (ART) [4], yet concerns about their Quality of Life (QoL) have risen. HIV-related stigma has been found to be a key stressor affecting QoL of these youth [1,5]. Although several studies have elaborated how HIV-related stigma undermines the HIV management cascade for youth [6–10], little attention has been devoted to the lived experiences of YLWHA with HIV-related stigma and its effects on their daily life especially in the Ugandan context.

Studies of HIV-related stigma among YLWHA is of relevance since these youth are highly vulnerable to stigma [11] and their number is on the rise. Youth’s vulnerability to HIV-related stigma is exacerbated by social and economic marginalization [12], as well as the rapid physical and psychosocial transitions. Stigma often follows the fault-line of this existing social marginalization and tends to magnify and perpetuate it [13]. This is compounded by the general lack of youth-friendly programmes to enable youth to navigate through HIV-related challenges [14]. Additionally, since youth are in initial stages of cognitive, physical, and social development, they may be exposed to, interpret, express or react to HIV-related stigma in different ways from adults. For instance, HIV-related stigma has been reported to evoke maladaptive responses in youth such as sex and substance abuse [15]. Youth also have less control over their living situations due to their high dependence on adults/caregivers and are often less aware of their rights compared to adults [11]. Moreover, they suffer unjustified social blame for the infection whose genesis they often had no control over (in case of perinatal infection and sexual violence) [11]. Adding to the age-group-specific construction of stigma, socio-cultural contextualization is also at play. It has been documented that variations in the extent, effect and nature of HIV-related stigma occur across social ecological contexts [13, 16]. Hence, examining how this stigma presents in a particular context such as homes, school and larger community is crucial for a deeper understanding [13].

Several theoretical perspectives have been advanced to define and explain the cause and nature of HIV-related stigma. Most of these draw basis from the stigma theory proposed by sociologist Erving Goffman. Goffman conceptualized stigma as a socially and contextually constructed attribute that is deeply discrediting to an individual, creating a devalued deviant identity in the eyes of society for those possessing it [17]. Accordingly, a stigmatized person is one who bears an undesirable difference. Deacon provides a simplistic definition of HIV-related stigma as “negative things people believe about HIV/AIDS and People Living With HIV/AIDS (PLWHA)” [18]. Other definitions have been proposed to include enacted stigma like discrimination [19]. Earnshaw and Chaudoir argue that the individual experiences and effects of HIV-related stigma are determined by the way social mechanisms of HIV/AIDS impact individuals including those without HIV/AIDS (stigmatizers) [20]. As postulated in this model, HIV/AIDS is a socially devalued mark that elicits peoples’ reactions when it ensues in society. For the HIV-uninfected, their cognizance that deviants exist, threaten their health and may possess moral blemishes [21], evokes prejudice, stereotypes and discrimination resulting into HIV-related stigma enacted for the HIV-infected. The reactions of the HIV-infected are psychological processes built on the knowledge that they are social deviants who may have violated social morals and are subject to other peoples’ negative treatment. They therefore experience internalized, anticipated, and enacted stigmas with several deleterious outcomes. Internalized stigma is when the HIV-infected believe the devaluing attitudes from society towards them [22] while the expectations by the HIV-infected of stereotypes, prejudice and discrimination from the HIV-uninfected leads to anticipated stigma [23]. On the other hand, when prejudice is behaviorally directed towards the HIV-infected by the HIV-uninfected, enacted stigma occurs [20]. We adopt this HIV-stigma model since it enabled us to study both the stigma mechanisms and their outcomes.

Given the breadth and detrimental impact of HIV-related stigma documented in the international literature [24–29], there is need for a clear understanding of the stigmatization process and lived experiences of YLWHA with HIV-related stigma, that can drive contextualized stigma reducing interventions. In the present study, we pursued to understand lived experiences and effects of HIV-related stigma by answering the research question: how do YLWHA in Western Uganda experience HIV-related stigma and its effect?

## Methods

### Study design, settings and participants

The study drew on photovoice methodology as described by Wang and Burris [30]. This methodology has been found to be effective at describing personal experiences, analyzing concerns in the community, synthesizing solutions to problems faced by marginalized people such YLWHA [30, 31] and at facilitating advocacy for such groups [32]. Hence, it moves away from established politics of representation by experts and allocates choice and control over representations to those it concerns [33]. In photovoice, photography is used to stimulate experiential expression, to access people’s inner worlds and convey their experience of the world [33].

We purposively selected 11 YLWHA from an existing Peer Support Group (PSG) at a regional referral hospital in Kabarole district, Western Uganda. The PSG, a mixed group of girls and boys of various ages who are HIV positive, meets every Saturday morning in a shelter close to the ART clinic. One health worker oversees the hospital-based activities of this group that involve sharing experiences, peer counselling, and health talks from the in-charge health worker or other resourceful people. Participants’ inclusion for the photovoice project was based on age (12 to 19 years), regular membership to the PSG, being able to speak either the common local languages (Luganda & Rutooro) or English, and willingness to participate over a period of 5 weeks. Exclusion criteria were other stigmatized health conditions such as tuberculosis disease and physical disabilities, as these could confound experiences of HIV-related stigma.

### Procedure

After explaining the study purpose and procedure to the ART clinic in-charge, we were referred to the PSG in-charge who aided recruitment of participants. During one of their meetings, the PSG in-charge identified and referred to us potential participants based on our inclusion and exclusion criteria. In a quiet private room at the ART clinic, we explained the purpose and procedure of the study to each potential participant and sought their consent/assent. We recruited 11 participants in the age range 15–19 years, including 6 girls. We conducted 5 weekly sessions, each lasting 2 ½ hours on average, in the months of September and October 2018. The sessions were facilitated by the Principal Investigator (PI), who is fluent in English and a native speaker of Luganda, and an assistant who is fluent in English and a native speaker of Rutooro. These facilitators’ immense experience in working with YLWHA enabled them to establish rapport with participants and to create a conducive atmosphere. The sessions were held in the same venue and following the weekly PSG meetings. Discussions were conducted predominantly in Rutooro (a local language of the area) although participants occasionally switched to Luganda and English.

The first session was aimed at discussing the meaning of stigma, the goal of the study, the photovoice procedure and the ethics of photography. This session enabled us to incorporate participants’ input into the final protocol. For instance, hand drawn pictures were suggested by participants and they also chose pseudonyms. In the second session, participants were challenged to remember HIV-related stigma experiences and their reactions, and to visualize such experiences in their received notebooks as hand-drawn pictures. Each participant then briefly discussed the drawn pictures with the PI or assistant for guidance. This activity developed a skill in participants to transform HIV-related stigma experiences into visual images that was required during photography. In the third session, we provided a digital camera to each participant. After being taught basics of using a camera, reviewing ethics of photography as well as personal and camera safety, participants practiced around the study site under close supervision. They were then tasked to take photos to portray their experiences of HIV-stigma and the effect of this stigma in their community for a period of one week. In the fourth session, participants selected at least 5 photos they wanted to discuss with the researchers and their peers. They then wrote narratives and captions for their five photos individually in their notebook. Two boys had trouble writing and they were duly assisted by the research assistant. In the fifth session, a group discussion was organized. One at a time, participants discussed their photos while allowing others to comment, supplement or ask questions as they reflected on the photos, narratives about them and their own experiences. The audio recorded discussions followed a modified root-cause questioning guide ‘SHOWeD’ as described by Wang [31] and outlined below.

What do you *Show* in the photo?

What is really *Happening*?

How does this show *Our* experience with HIV-related stigma or the effect of stigma?

*Why* does this problem exist?

What can we *Do* about it?

### Information sharing

Following the discussion of their photos, we prompted participants to suggest how their photos and stories could be used to convey their plight to the wider community, practitioners in HIV/AIDS care and policy makers. Through consensus, they agreed that the following activities could be done;

1. To exhibit the photos and the stories in the annual regional youth conference and in one of the major towns in the region. A photo-text PowerPoint exhibition was organized during the youth conference in which participants voluntarily attended though they choose not to participate in the presentation. However, they were active in the discussion that ensued like any other conference attendees. A photo exhibition in the town had not been organized by the time this paper was submitted.
2. To present findings of the project in one of the regular seminars organized by the Community of Practice (CoP) on young people living with HIV/AIDS in Rwenzori region, western Uganda. The CoP is a consortium of civil society organizations, community based organizations, non-governmental organizations and policy makers in the region. Members of this CoP meet periodically during seminars, workshops and other forums to create common knowledge on how to improve the welfare of young people living with HIV/AIDS. The findings were presented in one CoP seminar in which participants voluntarily attended. Two authors EK and KMJ presented the findings and participants contributed to the discussions like other people in attendance.
3. To print photo-text books to be distributed to schools within the region in order to stimulate debate in schools around the issue of stigma and discrimination. By the time of submission of this article, this activity had not been done.

### Ethics

The study protocol was reviewed and approved by the Research Ethics Committee (REC) of the AIDS Support Organization (TASO) in Uganda (reference number, TASOREC/009/18-UG-REC-009) and the Uganda National Council of Science and Technology (reference number SS 4587). We obtained informed written consent/assent from all participants and informed written consent from parents/caretakers of 7 participants who were minors. These parents/caretakers were invited by phone calls to the research site. We also requested each participant to sign a photo release form that granted us permission to use the photos they had taken. Pseudonyms were used throughout the audio recordings, analysis, reporting of results and all public exhibitions. All data were secured with a password in the personal computer of the PI. We reimbursed each participant with an equivalent of 7 USD to cover transport costs for each of the sessions. After the last session, each participant received a photo album containing personal photos they had taken as an appreciation for their participation.

### Data management and analysis

Preliminary analysis begun during data collection when potential themes to be captured in the photographs were identified jointly with participants. This occurred during the participant-facilitator discussions of hand drawn pictures while reflecting on the jointly agreed meaning of stigma. Participants described the type of stigma experience e.g., mistreatment, fear, loneliness, isolation, etc. and outcomes of stigma that their hand drawn pictures depicted. They then reflected on each other’s visualizations and narratives, and sought for shared experiences and common themes while being invited to also notice particularities in their experiences with stigma. In the final analysis, all audio materials were transcribed verbatim and all necessary translations to English were done by a postgraduate students fluent in the local languages. The translations were checked against the original audio recordings and texts by the PI for accuracy. Data analysis was grounded in phenomenological hermeneutics and involved a multidisciplinary team of 5 researchers (EK, JD, DR, SV and AE). We followed the philosophical underpinnings of Ricoeur [34] and van Manen [35]. With phenomenological hermeneutics, researchers use interpretive activity to make participants’ experiences comprehendible to their audience leading to dual interpretation [36]. Within-case and cross-case analysis was performed on all transcripts of the group discussions, photographs with their captions and stories penned in the personal notebooks. In the analysis we employed the hermeneutic cycle [37].

All researchers individually read repeatedly through the written data to get immersed and maintain an emic stance to participants’ experiences. We then made notes about our understanding, observations and reflections of participants’ narratives. Later, all photos with their captions were displayed, closely observed, and annotations were made by all researchers independently. In doing so, we continued to document our reflections and interpretations inductively and as close to the data as possible. For instance, when a participant said ‘I am always left at home alone to do all the house chores when other children go to school’, we condensed two meaning units from this: ‘overburden by tasks at home’ and ‘denied chance to go to school’. We then transformed our individual notes as a group into potential analytical themes by formulating concise phrases with minimal abstraction. For example, the two reflections above were both interpreted as ‘experiencing occupational injustice’. Each identified potential theme was discussed in the group to ascertain how well it interprets participants’ experiences. Extracts of texts and photographs related to each theme were clearly marked to maintain an audit trail. We then looked for connections between themes, collated them in an iterative manner basing on conceptual similarities to form clusters (overarching themes) and provided a descriptive label for each cluster as shown in Table 1 below. For example, the theme ‘experiencing occupational injustice’ was clustered under the overarching theme ‘experiencing injustice’. Finally, EK organized all the data, themes and overarching themes in a computer software NVIVO version 10 for easy retrieval.

**Table 1:** Main themes and themes identified from the analysis.

Main themes	Themes
Being devalued	Being treated as waste
	Negative self-image
	Negative societal attitude
	Neglected
	Changing social environment
	Taking up meaningful roles in society
	Concealing their status
Experiencing fear	Fearing death
	Being hyper vigilant
	Being highly visible
	Being highly sensitivity
	Fearing to disclose their status
	Fearing medicine
Experiencing injustice	Occupational injustice
	Unjust treatment mainly at home
	Confidentiaity and privacy
Lacking Future perspectives	Life on hold
	Death as expected outcome
	Intrusive negative thoughts
	Redefining goals and future
Feeling lonely	Self-isolation
	Exclusion and seclusion by others
	Un-answered questions

## Findings

### Demographics

We involved 11 YLWHA including 6 females, aged 15–19 years (mean age = 17, SD = 1.34). Majority of them (82%) were perinatally infected, 1 reported getting infected through rape and 1 was not sure of the source of infection. Only 3 of the participants were still attending school and the others had dropped out. All participants had lost at least one parent.

The five interrelated overarching themes are presented below basing on the breadth with which they cover participants’ experiences.

### Being devalued

The youth experienced social and self-devaluation due to their HIV seropositive status. In their narratives, they posited that HIV is considered a strange disease in the community because historically it had no cure and it is associated with shame, long suffering and death. It further appears strange when only one member in the family has it or when all the family members bear the infection. Such occurrences breed skepticism in people, including the infected, that scientific explanations struggle to address. All participants alluded to the fact that people are ignorant about HIV/AIDS and that those who read or hear about the disease do not understand it, rendering it a mystery that arouses prejudice and stereotypes. As a result, the youth experienced that when their status was disclosed to others, it changed the way others perceived them. All youths were distressed by how they are seen as alien to community, cursed, abnormal and different. They were always mindful that they are seen as dangerous and a burden to others.

These views often evoked differential treatment and mistreatment in homes, schools and broader communities. Most participants shared accounts of parental neglect and abandonment. Parents reportedly lost the love and care for these youth on learning that they had HIV and relegated their responsibility to caretakers, mainly grandparents. This parental abandonment was seen as a way of avoiding their own status from being involuntarily disclosed through their children in cases of perinatal infections as well as avoiding the social view of their children’s uselessness projected onto them.

**Fig 1:**
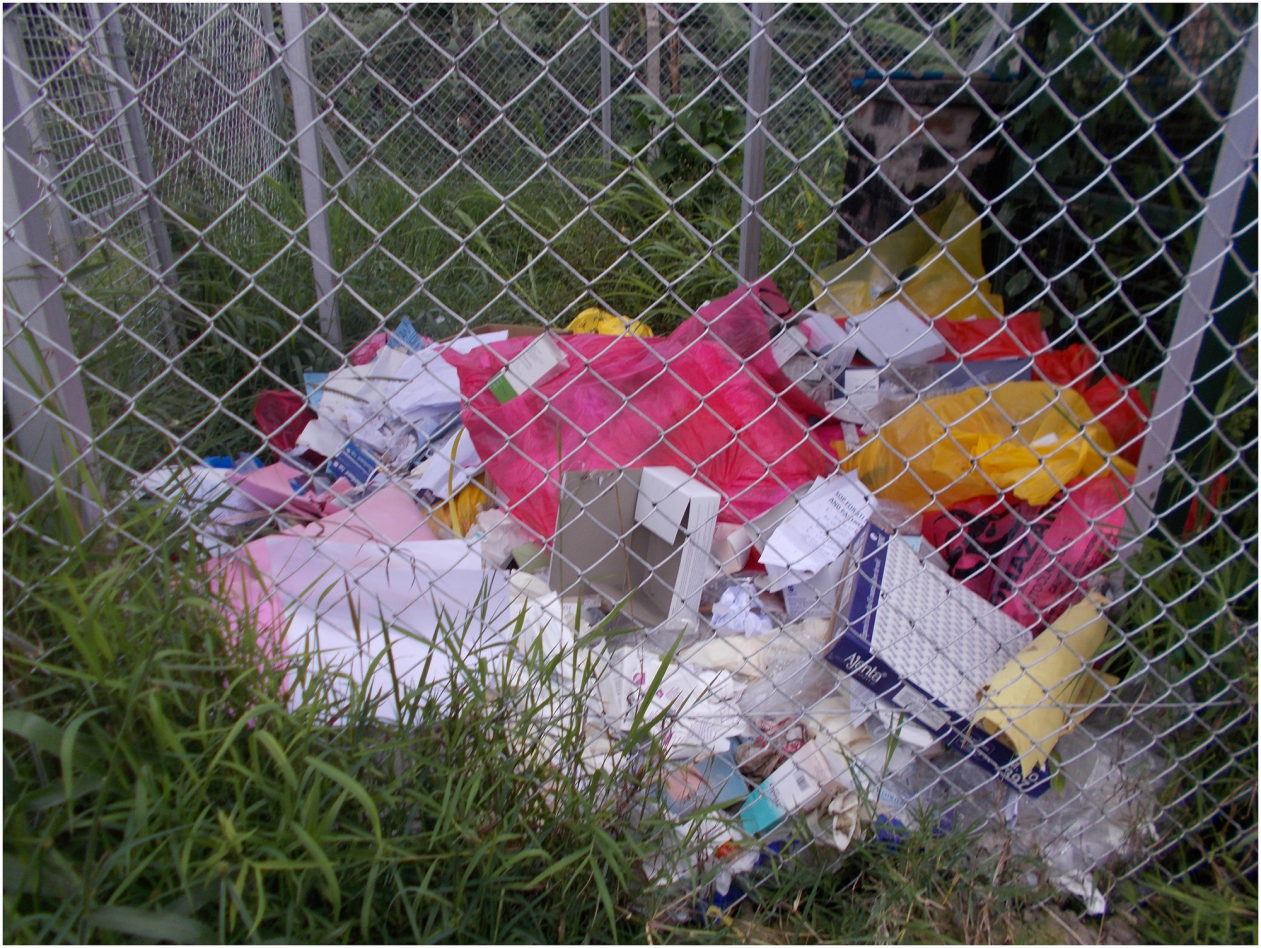
“**People view us as useless rubbish that should be separated from the rest of the people**. I think they are waiting for us to die so that they can have their space. This starts with our own parents because they know that their status can be revealed through us. So, like they do not want us to be in public yet they are the ones who gave us the disease”.

In many instances, they were seen and treated as worthless beings who did not deserve further investments since they were considered unhealthy and expected to die early. Several youths represented in their photos how they felt treated as trash and one described this as being worse than the way animals are treated. A leading threat through these youths’ experiences with stigma was thus that it disvalues their lives and makes them feel subhuman. They also started to believe that they were useless and expected others to treat them as such. In their accounts, many depicted a sense of not owning their lives and carrying a huge burden of life on their shoulders. This made them to think that life was not worthy living and some consequently resented medication. Suzan, a 16-year-old girl expounds on this experience in her photo and accompanying caption (Fig 1).

Mafene, a school going boy also depicted and narrated the deep negative thoughts the devaluation evoked in him:

“*At school the teacher onetime said that the cure for HIV is death*. *Whenever I pass such materials [bricks*, *sand and stones] at construction sites*, *I do not think about building my house but I think of how my grave will be constructed*”

(Mafene, 16-year-old boy).

Youths’ narratives testified of how they actively coped to avoid or tackle this stigma. The insulting, teasing and bullying at home and in schools caused some to move from one caretaker home to another in the quest for better life, and others to change school or dropout. This depicts the need for many of these youth to break out of the realm of stigma and see themselves in a more positive perspective, through seeking more valuing surroundings and assimilate this in their self-value. Some emphasized that concealment of status was warranted in social settings, to avoid undue attention and devaluing treatments that cause such negative thoughts and low self-value to emerge. Others emphasized that to overcome the negative societal views and attitudes, their individual effort is necessary. They experienced how openness about their status denied rumors and gossip room and instilled a sparkle of change in their social environment. They additionally called upon others in the fight against HIV-related stigma to sensitize communities with correct information in public places such as schools and worship centres. Others pursued to be a role model for peers with HIV in their community. In further exploring the meaning of this activism, we understood that participants sought a meaningful role for themselves in relation to others with HIV and to their community, which was a significant source for retaining or regaining self-value. As another way of improving that, participants argued that they needed to actively and consciously elevate concerns about their life and health above what others think or say.

### Experiencing fear

**Fig 2:**
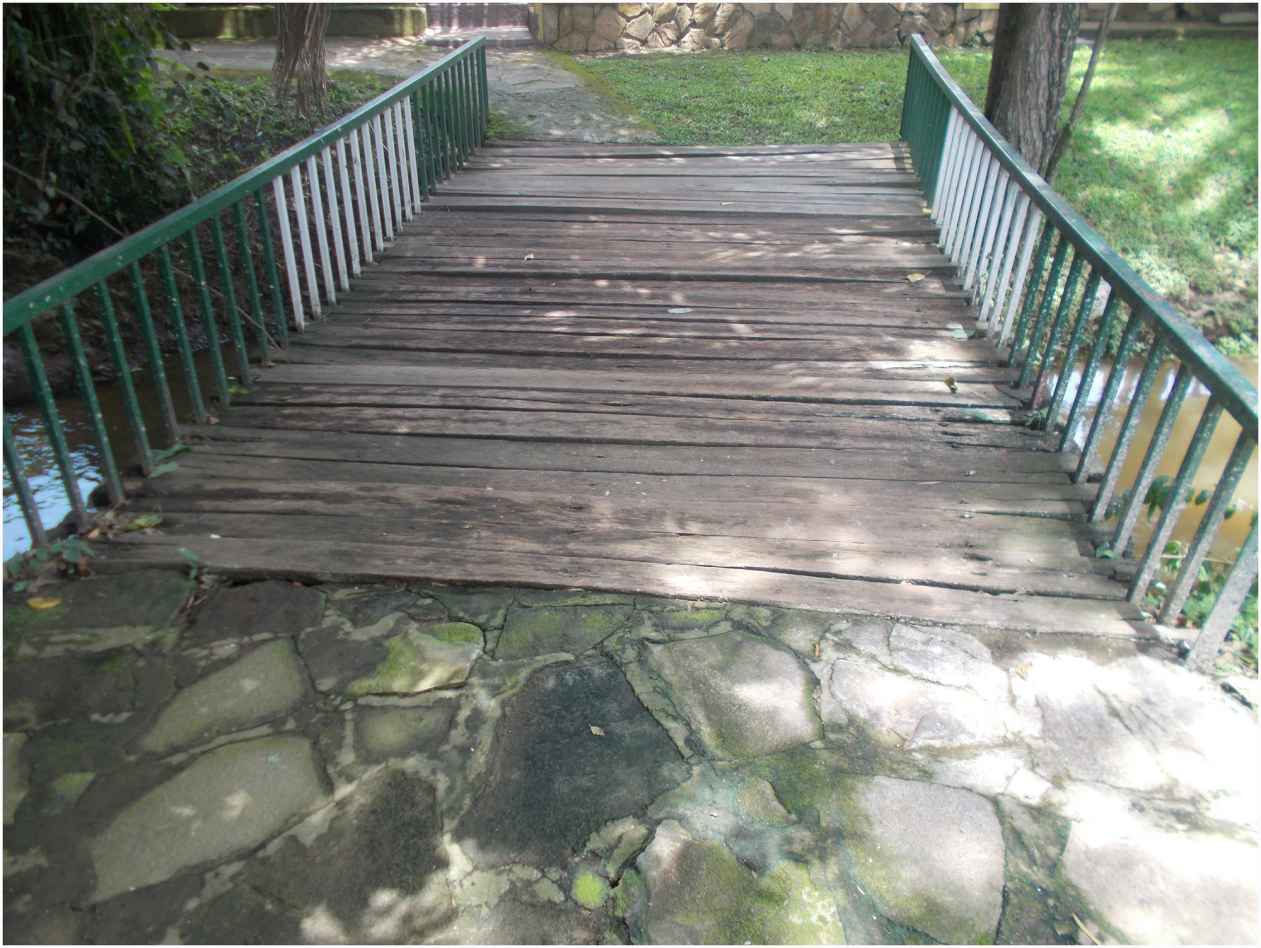
“I always pass here when going to the health centre but each time I fear that these rotting pieces of wood will break and I fall in the stream just like I fear that people will one time get to know my HIV status as I interact with them”.

Participants’ discourses were dominated with fear and this strongly affected their life with HIV. Both voluntary and involuntary disclosure of status were highly dreaded due to the threat of HIV-related stigma. In-box, a 19-year-old boy, portrayed and narrated deep seated fears of status disclosure (Fig 2).

Consequently, many youths felt that they had to live with a secret and were always on guard to maintain secrecy. Physical pointers of HIV like body rashes, persistent cough and weight loss bothered them a lot since they are recognized signs of HIV/AIDS in their community. Almost all participants reported disguising their medicine not to trigger suspicion. Prity, a 15-year-old girl, narrated how she used to keep her medicine in sanitary pad packs, hoodwinking others to think that she was keeping used sanitary pads and therefore to avoid touching them. Their hypervigilance, however, was sometimes a basis for others to be suspicious and to institute investigations, especially in boarding schools. Many youths shared the experiences that others were afraid to live with them, thinking that they would contract the infection. While unfounded, it affected their social interactions. Participants seemed to internalize this fear and similarly felt that they were a threat to others as portrayed in their photos and stories. They were highly sensitive to others’ actions, always suspecting them of gossiping or scheming evil for them, as Ricky narrates:

“*I was in school and wherever I would step*, *friends would all keep quiet yet before they saw me*, *they had been laughing and enjoying*. *I would keep asking myself why they would keep quiet whenever I approached them*. *I felt bad and thought of first leaving school then I would go back to school in future when I am okay*”

(Ricky, 15-year-old girl).

Being dependent on medicine was another prominently depicted aspect of their lives surrounded by stigma-induced fear. Despite their unanimous awareness that medicine was a resource for a healthier long life, they viewed it as a huge burden. They detested medicine and they felt branded by society as ‘those on medication’. To know that they must take medicine for life was emotionally taxing. They were additionally concerned that they had to take many tablets a day and that the tablets were also big, literally and figuratively. Angella, a 16-year-old girl, narrated how she experienced the fear medicine elicits in others and herself:

“*I used to carry my drugs from the clinic in the tins*, *as others would leave the tins at the clinic*, *until a boy on the street told me that my “big drug” [HIV drug] was making noise and scaring him*”

(Angella, 16-year-old girl).

**Fig 3:**
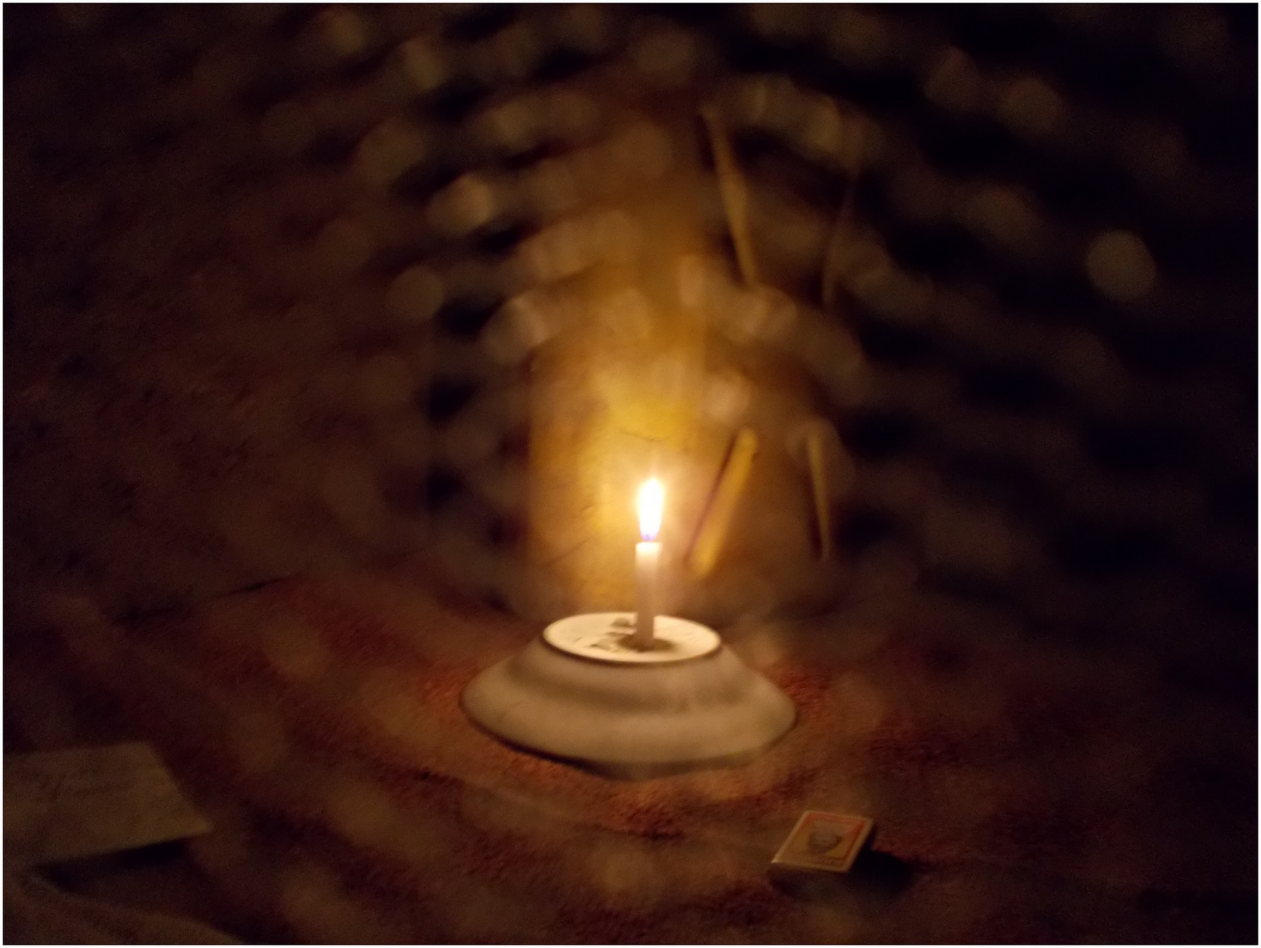
“**Because people tell me that I can die anytime, I fear darkness and I have to light a candle every day before I sleep**. This also is very dangerous because one time I almost got burnt while asleep, yet I stay alone in my room”. (Dagie, 17-year-old boy).

ART medication prolonging life notwithstanding, participants were moreover preoccupied with the fear of death due the social perceptions on HIV/AIDS as a deadly condition they internalized. They therefore believed that they were candidates for death anytime, as depicted in the photo and narrative of Dagie, a 17-year-old boy (Fig 3). They stated that anything that reminded them of their status also made them worry about death.

Narratives above depict fear as a dangerous experience of living with HIV/AIDS among these youth since it affects different domains of their life such as emotional wellbeing, physical health and relationships. To overcome the fear, many suggested taking good care of themselves to improve their physical health in order to project a good image in society.

### Experiencing injustice

Participants experienced HIV-related stigma in several situations in which their rights were violated by others at home, school and broader community. They were reportedly denied equal chance to enroll or stay in school, interact with other people, and to learn new things. Although many found schooling challenging, the choice to dropout was made by their parent/caretaker who felt that they were unfit for school based on the health condition of these youth and the prejudicial social views already described under preceding themes. As a result, they were locked indoors and turned into slaves. Within these homes, they decried being subjected to disproportionately excessive domestic chores that involved cooking, cleaning, laundry, and looking after animals. Unique Unice’s account in the quote below is illustrative of the distress she felt as she struggled with the injustice brought about by HIV-related stigma in her family, as shared by many participants. Participants felt getting overwhelmed by such demands and voiced concerns of how they would manage given their flimsy health.

“*I was made to do all the house chores at home while other children go to school*. *They just made me a maid to work for others*”

(Unique Unice, 18-year-old girl).

**Fig 4:**
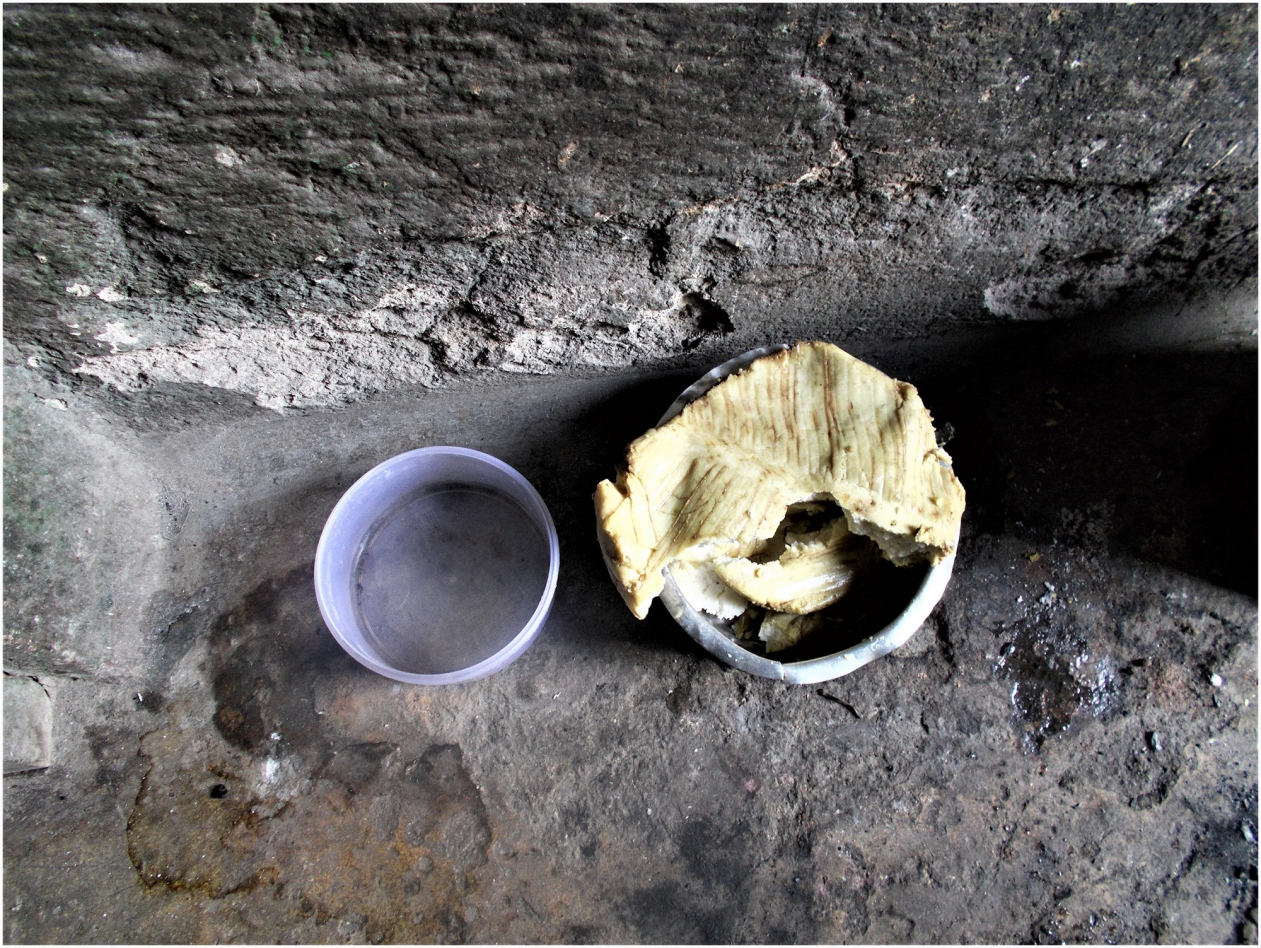
“**This is the type of food I was always made to eat by my stepmother when others were eating better food**. You know I had to swallow drugs and with such food it was not easy to continue with my medicine until when I left that home” (Small, 17-year-old girl).

In addition to the experience of occupational injustice, domestic HIV-related stigma was also experienced through being denied basic needs such as food and clothes. Some reported being fed on leftovers and sometimes spoilt food and having to eat alone while others were sharing a plate. They also reported staying in horrendous situations within the home. Small, a 17-year-old girl, felt the injustice when she was made to eat poor quality leftover food as others at home would feed on better fresh food as shown in Fig 4 and corresponding caption below.

In a few cases, physical assault was reported, especially by those who lived with stepmothers. For some, stigma-induced injustice was also felt in teachers disregarding their right to privacy by publicly disclosing their status and humiliating them. The consequent disgust and hatred for teachers and schools that this evoked in some participants, was clearly noticeable in their accounts and strongly marked how they experienced living with HIV. Josh, 18-year-old, school going boy provided a representative narrative for this experience:

“*My teacher saw me at the ART clinic and later disclosed to my classmates that I am HIV positive and on drugs*. *I felt very bad and I tried to drown in this pond [depicted in a photo] but it was too shallow*. *After this attempt an inner voice told me to be strong*. *I will never forgive that teacher in my life*”

(Josh, 18-year-old boy).

Participants experienced that whenever they rose up to contest such injustices especially within homes by voicing their concerns, a positive change was realized. They also suggested reporting some of the injustices to significant people in their community like health workers and local council leaders to effect some change.

### Lacking future perspectives

**Fig 5:**
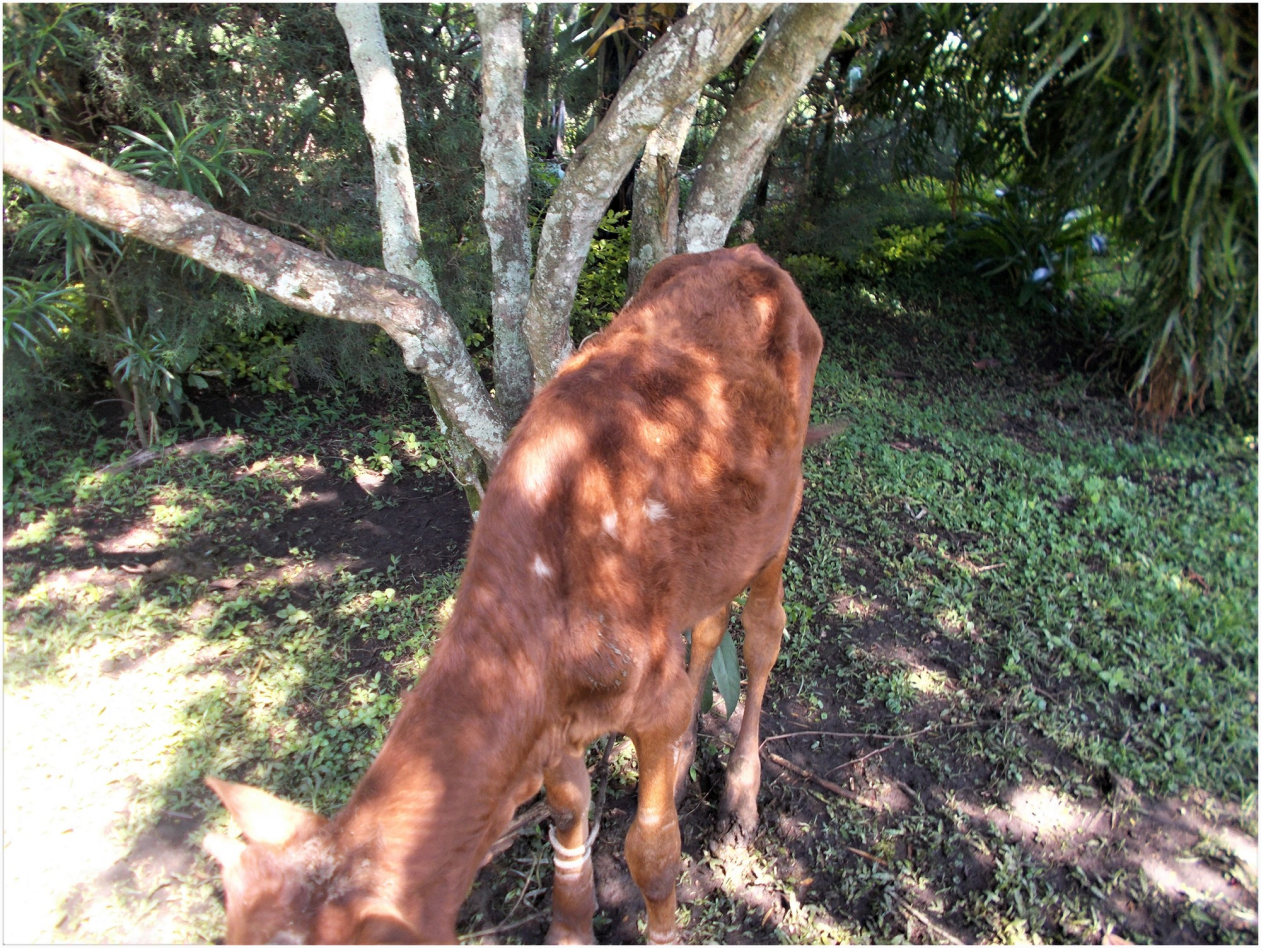
“**Like an animal tied on the tree, you cannot go further in your plans**. You just keep there, and you do not enjoy life” (Suzan, 16-year-old girl).

Participants looked at their future as unfeasible due to their status and the associated stigma around it in their surroundings. Since death was seen as an unavoidable eminent outcome for them, parents/caretakers and teachers reportedly found it unnecessary to invest in these youth and just waited for the day they would die. The participants shared multiple accounts revealing their awareness of how that imposed limitations on their future outlooks and aspirations. Despite the desire to have a family in the future, they were aware of stigma-related challenges to get into a romantic relationship. They additionally noted that the social devaluation attached to their health condition could not guarantee good education for them and therefore a good job in future. They therefore portrayed their life as being on hold and as a result they revealed having developed intrusive negative thoughts. Their accounts testified of their sorrows, sadness, regrets for being born and suicidal ideations. Suzan, a 16-year-old girl, represented her lived experience with the impact of stigma on her future as ‘being tied’ (Fig 5).

Some youths’ accounts showed how they had struggled to redefine their future goals and aspirations, alongside the mourning over missed opportunities in life. They emphasized that accepting their medical and social status and learning to care for themselves were the first steps towards achieving their goals. They were aware of challenges to achieve this, but they noted that they needed to be creative to do whatever job was available to earn an income. Changing school was perceived by many as necessary to avoid dropping out. They also valued psychosocial support from others to raise their hopes to work and attain their future goals.

### Feeling lonely

A high tendency to isolate themselves was evident in participants’ stories. Some stated that they were expected to separate themselves from others to avoid contaminating them. They often did this unwillingly in fear of reprimand from caretakers. Voluntary self-isolation was also frequently framed as a survival strategy to maintain secrecy of their status, as reported earlier. Even in the presence of others, YLWHA experienced mental isolation and loneliness. They were always lost in thoughts and as a result, unable to resonate with others in discussions. Such deep thoughts were understood to arise from a few but pertinent questions that they always had. Their stories were punctuated with questions like; why they are the only ones with HIV in their families and how exactly they could have got this infection. Josh, a 19-year-old boy, narrated his experience of loneliness and how he was affected by it while still at school in the quote below.

“*Instead of reading my books I would be deep in thoughts about my health and future*. *I think this affected my performance in S4 yet I know I am a bright boy*”

(Josh, 18-year-old boy).

**Fig 6:**
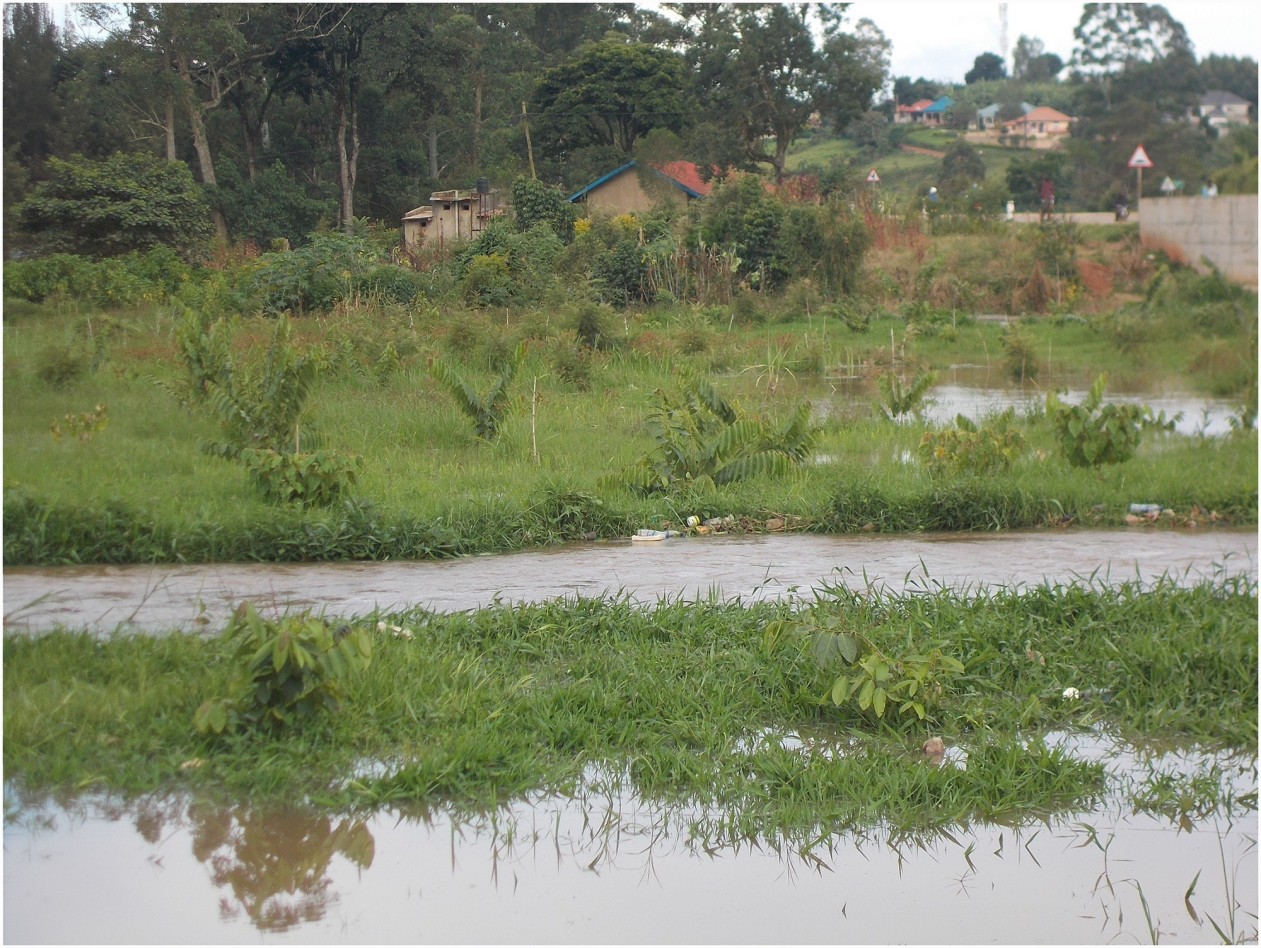
“**People used to throw me here and there like the water that flows continuously without resting**. Wherever I would go to stay they would tell me to go elsewhere, where I can fit. So, I would continue to move endlessly in such a bad situation. I stayed with a total of not less than four families and each kept sending me away” (Abooki, 19-year-old boy).

Discrimination seemed prevalent within direct networks of participants at home and school. They were always given separate rooms to live in at home and many appreciated this as a way of achieving privacy and further concealing their status. However, this was a clear seclusion of these youth since in their setting people prefer to live together even in small living spaces. These discriminatory tendencies at home were always heightened whenever the youth developed physical signs of the disease. Dagie, a 19-year-old boy, narrated how he felt isolated when his caretaker stopped him from playing, eating and sitting together with other children at home whenever he fell sick. Some experienced the extreme form of discrimination when they were thrown out of the homes by caretakers. Abooki, a 19-year-old boy, provides a photo (Fig 6) and a narrative of this experience and how it evoked a deep sense of loneliness:

## Discussion

This study shows a rich patterning of enacted, anticipated, and internalized stigma experiences of YLWHA and provides insights into the myriad sequels lived through by these youths. In their day-to-day lives, participants revealed being devalued, experiencing fear, lacking future perspectives, experiencing injustice and feeling lonely. Our results resonate with other studies on HIV-related stigma [38, 39] and theorizations of stigma [14, 18, 21, 40] in placing devaluation and discounting of people living with HIV/AIDS at the center of stigmatization. Consistent with Earnshaw and Chaudoir model [21], we found that the social devaluation and discounting was often endorsed and expected by YLWHA leading to internalized and anticipated stigma respectively. It thus became a foundation for the actions, attitudes, and stereotypes that characterized the life of these youth with HIV.

Enacted stigma presented as interpersonal discrimination, gossip/rumors, insults and bullying. These experiences were prevalent in homes, school settings and the community and they caused anguish in the lives of participants. Similar incidents have been reported in prior studies with YLWHA [11, 41, 42] and other groups of People Living With HIV/AIDS (PLWHA) [43]. As a result of stigma in these fundamental socio-ecologies [41], participants seemed to be caught between a rock and a hard place. Bullying in school was vividly reported as experienced by the school going participants, as also found by Abubakar and colleagues [42]. Our study added the understanding that YLWHA were prime targets due to their uniqueness at school, exhibited through self-isolation, daily medication and poor physical health. Despite their potential to change stigmatizing attitudes and transform societies [44], schools in these settings continue to exude high levels of stigma. This could relate to entrenched social views and attitudes that in-school and community sensitization and education programmes have failed to overcome. Moreover, in-school support programmes tailored for those living with HIV are lacking although provided for in the national HIV/AIDS policy for Uganda. This renders disclosure of status in school settings pointless. Additionally, much emphasis has in the recent past been devoted to primary prevention strategies as these are deemed fit for in-school youth. However, some of the preventive messages are implicated in causing stigma to the already infected youth [6]. School-based interventions are hence urgently needed to address HIV-related stigma and promote status disclosure for HIV-infected youth to receive appropriate support in schools. This can be achieved through continuous sensitization of all school stakeholders to overcome social prejudices towards PLWHA. This sensitization should be based on a contextualized understanding of the daily HIV-related stigma experiences, some of which this paper underscores. Our findings allude to this when YLWHA suggested being open about their status to deny others chance to gossip about them and to provide correct information about living with HIV/AIDS.

This paper highlights mistreatment, differential treatment and neglect by parents/caretakers as forms of injustice that YLWHA experienced daily. The story of Unique Unice, who was denied chance to go to school and turned literally into a slave at home, represents many similar experiences. This enactment of stigma raises pertinent questions regarding the safety and rights of these youth in homes. In the case of YLWHA, homes abrogate their inherent role of being supportive and protective social capsules for vulnerable members of society [45]. We understood this injustice as emanating from the already alluded to social devaluation and discounting that creates a feeling and belief in parents/caretakers that they labor in vain to raise a child who may die soon. Parents/caretakers foresee no future for these youth and gauge it unfeasible to invest their time and physical resources in them. This further disenfranchises these youth and perpetuates their dependence and vulnerability. Youth living with HIV/AIDS are profoundly affected by the enactment of stigma at home due to power disparities caused by their dependent status in homes [46]. For majority, their orphan status additionally makes them more vulnerable to abuses from caretakers with impunity since they cannot challenge the status quo [47]. In a family context, enacted stigma is detrimental to YLWHA since they have no advance choice of family to belong to [48] and because families are supposed to be sources of unconditional love. They therefore feel sternly betrayed by individuals they trust to support, care and love them. This stigma threatens a fundamental need to belong [49]. Undeniably, the story of Abooki, who kept changing caretaker homes clearly illustrate how HIV-related stigma can cause homelessness in YLWHA. Since these youth are vulnerable and marginalized, advocacy needs to be built around them by significant members in society such as healthcare workers, teachers, and local council leaders to shield them against abuses through educating and supporting their parents/caretakers. These youth should also be empowered to open up against injustices at home and elsewhere by confiding in their advocates. This empowerment can be provided at school by teachers and at health centres by their counsellors and medical staff. Impromptu home visits could be organized by these advocates to support caretakers and to ensure that these youth are not abused. Home visits in HIV care have been reported to improve retention in care and medical adherence [50, 51] and could therefore take up an additional mandate to ensure that YLWHA live in a stigma free home environment. This approach, however, needs to take keen note of the social structural challenges of power disparities discussed above. It is likely that YLWHA might be afraid of reporting their caretakers and further severing the already fragile bonds with them. In African contexts, family ties tend to alienate involvement of outsiders [52] and this is one of the reasons why cases of domestic violence can flourish unreported [53].

Being and feeling lonely was another set of experiences that painted the life of youth with HIV. The endorsement of societal beliefs and attitudes about HIV led YLWHA to believe that they were useless, had no future since they were to die soon and that they were a threat to others. This internalization of stigma triggered voluntary self-isolation in YLWHA as a strategy to conceal their status and escape discrimination and other stigma enactments or involuntarily isolation as required by their caretakers. This finding is in agreement with those of other studies [41, 54, 55] regarding self-isolation of YLWHA. Loneliness has far reaching negative effects to the psychosocial wellbeing of all PLWHA such as depression and lack of support [56], but for youth, it is such a dilemma since they may lack appropriate mental processes and capacities to challenge entrenched societal views upon which stigma becomes internalized. In addition, belonging to peer groups is typical of this age-group [57]. Based on our findings we advocate for continuous counseling of YLWHA that empowers them to challenge societal views and attitudes and education for all people to provide correct information about HIV/AIDS and to dispel myths. Prior studies point to correct information about HIV as a major determinant for attitude change [58], but deep-rooted culture-specific attitudes may undermine these programmes. For instance, beliefs in the supernatural as a cause of HIV/AIDS eminent in this context may counter etiological facts about the disease. In a review by Brown and colleagues, education and sensitization interventions resulted into superficial tolerance of PLWHA with no guarantee of effect in actual encounters [59].

Lastly, we found that YLWHA experienced fear on a daily basis. Stories of Dagie and Mafene demonstrate and represent several ways in which fears of death present daily in the lives of these youth, despite the assurance that with ART they can live longer. Just like extant literature [60, 61], our findings relate this fear to the historical prognosis of HIV during the pre-ART era. Therefore, sensitization and reassurance, is necessary to dispel such historical views. In the HIV care cascade, health workers and other care providers deliver this sensitization but the involvement of those who have lived with HIV for a longer time can be of added value since they face similar daily experiences and YLWHA can easily relate with them. Fear of revealing their status also evoked a lot of distress on a day-to-day basis due to anticipated stigma. Voluntary and involuntary disclosure were thus diligently avoided as a way of evading enacted stigma. Disclosure concerns for PLWHA have been widely studied [12, 62, 63] and literature reports disclosure as having a dual effect on the wellbeing of PLWHA [64]. On one side, it leads to psychosocial support and improved treatment adherence, while on the other, it leads to stigma. Due to this ambivalence, disclosure remains a challenge [65]. The story of Inbox in this study represents this fear of status disclosure and clearly portrays it as lifelong fear. We found that secrecy was preferred by YLWHA as also found in other studies [1]. Although a desired strategy to avoid enacted stigma, secrecy has been found less effective for YLWHA in school and home settings because of several revealers that can easily be decoded by people who stay close to them for a longer time [6]. For instance, ART medication often constrained secrecy in school and at home. Medicine was reported to unveil the status of YLWHA and to elicit stigma despite its reputation for prolonging healthier lives. We report ART medication as a perceived cause rather than a panacea to HIV-related stigma in youth, contrary to other studies [66]. Our study findings show that youth were willing to tradeoff medication to escape stigma at the opportunity cost of their health. This illustrates how concerns about the perceived social image of youth prevail over their healthy choices [67]. Further research is necessary to illuminate the medication-stigma causal relationship in HIV care and how this affects medication adherence and quality of life for YLWHA. Our findings indicate that the hyper vigilance to conceal their status further broached internalized stigma and made the youth hypersensitive to actions of others around them.

Even though HIV stigma has predominantly negative effects on the youth as portrayed in this study, protective factors and avenues to strengthen these youths’ resilience were proposed by participants. Further research that advances the knowledge on resilience in YLWHA is needed.

### Study strengths and limitations

We selected members of a PSG who were familiar with each other and interacted freely. As such, they were able to offer each other support. The use of photography enhanced storytelling, a practice enshrined in African traditions [68]. The study however had some limitations. First, participants were selected from a hospital in a fairy urban setting. We believe that HIV-related stigma experiences of those living in rural areas would differ from those in our sample. Second, being part a PSG, our participants may not adequately represent other YLWHA who do not belong to such groups since the PSG could have enabled these youth to better cope with or avoid stigma.

## Conclusions

The study illustrates youths’ experiences with HIV-related stigmas in different socio-ecologies. Prominent in this study is the enactment of stigma in homes and schools which ought to be significant settings for the safety and welfare of youths more especially the vulnerable ones such as YLWHA. The findings also bring to light an eminent need to address HIV-stigma by focusing on both the stigmatized and the ‘stigmatizers’ through interdisciplinary interventions that appeal to different QoL domains. The involvement of all stakeholders in the lives of these youth is thus proposed for wholistic interventions. Such interventions ought to recognize the contextual causes and lived experiences of HIV-related stigma among YLWHA in order to deliver substantial sustainable results.

## References

[1] Fielden S. J., Chapman G. E., & Cadell S. (2011). Managing stigma in adolescent HIV: silence, secrets and sanctioned spaces. Culture, health & sexuality, 13(03), 267–281. 10.1080/13691058.2010.525665 21049313

[2] Bekker L. G., & Hosek S. (2015). HIV and adolescents: focus on young key populations. Journal of the International AIDS Society, 18(2Suppl 1).

[3] Landefeld C. C., Fomenou L. A., Ateba F., & Msellati P. (2018). Prevention of Mother-to Child Transmission of HIV in Yaounde: Barrier to Care. AIDS care, 30(1), 116–120. 10.1080/09540121.2017.1390540 29034724

[4] Rutakumwa R., Zalwango F., Richards E., & Seeley J. (2015). Exploring the care relationship between grandparents / older carers and children infected with HIV in south-western Uganda: Implications for care for both children and their older carers. International journal of environmental research and public health, 12 (2), 2120–2134. 10.3390/ijerph120202120 25689350 PMC4344715

[5] Kimera E., Vindevogel S., Rubaihayo J., Reynaert D., De Maeyer J., Engelen A. M., et al (2019). Youth living with HIV/AIDS in secondary schools: perspectives of peer educators and patron teachers in Western Uganda on stressors and supports. SAHARA-J: Journal of Social Aspects of HIV/AIDS, 16(1), 51–61. 10.1080/17290376.2019.1626760 31179837 PMC6567167

[6] Zanoni B. C., & Mayer K. H. (2014). The adolescent and young adult HIV cascade of care in the United States: exaggerated health disparities. AIDS patient care and STDs, 28(3), 128–135. 10.1089/apc.2013.0345 24601734 PMC3948479

[7] Bernays S., Paparini S., Seeley J., & Rhodes T. (2017). “Not taking it will just be like a sin”: Young people living with HIV and the stigmatization of less-than-perfect adherence to antiretroviral therapy. Medical anthropology, 36(5), 485–499. 10.1080/01459740.2017.1306856 28379042

[8] Kurth A. E., Lally M. A., Choko A. T., Inwani I. W., & Fortenberry J. D. (2015). HIV testing and linkage to services for youth. Journal of the International AIDS Society, 18, 19433 10.7448/IAS.18.2.19433 25724506 PMC4344538

[9] Kahana S. Y., Fernandez M. I., Wilson P. A., Bauermeister J. A., Lee S., Wilson C. M., et al (2015). Rates and correlates of antiretroviral therapy use and virologic suppression among perinatally and behaviorally infected HIV+ youth linked to care in the United States. Journal of acquired immune deficiency syndromes (1999), 68(2), 169 10.1097/QAI.0000000000000408 25590270 PMC4312477

[10] Martinez J., Harper G., Carleton R. A., Hosek S., Bojan K., Clum G., et al, and the Adolescent Medicine Trials Network, J. (2012). The impact of stigma on medication adherence among HIV-positive adolescent and young adult females and the moderating effects of coping and satisfaction with health care. AIDS patient care and STDs, 26(2), 108–115. 10.1089/apc.2011.0178 22149767 PMC3266519

[11] Singh, V., & Lata, S. (2018). A systematic review of HIV/AIDS related stigma among children and youth living with HIV.

[12] DeMatteo D., Wells L. M., Goldie R. S., & King S. M. (2002). The 'family' context of HIV: a need for comprehensive health and social policies. Aids Care, 14(2), 261–278. 10.1080/09540120120076940 11940283

[13] Parker R., & Aggleton P. (2003). HIV and AIDS-related stigma and discrimination: a conceptual framework and implications for action. Social science & medicine, 57(1), 13–24. 12753813 10.1016/s0277-9536(02)00304-0

[14] Tylee A., Haller D. M., Graham T., Churchill R., & Sanci L. A. (2007). Youth-friendly primary-care services: how are we doing and what more needs to be done?. The Lancet, 369(9572), 1565–1573. 10.1016/S0140-6736(07)60371-7 17482988

[15] Swendeman D., Rotheram-Borus M. J., Comulada S., Weiss R., & Ramos M. E. (2006). Predictors of HIV-related stigma among young people living with HIV. Health Psychology, 25(4), 501 10.1037/0278-6133.25.4.501 16846325 PMC2392891

[16] Deacon H. (2005). Understanding HIV/AIDS stigma: A theoretical and methodological analysis. HSRC press.

[17] Erving G. (1963). Stigma: Notes on the management of spoiled identity. New York: A Touchstone Book Published by Simon & Schuster Inc.

[18] Deacon H. (2006). Towards a sustainable theory of health‐related stigma: lessons from the HIV/AIDS literature. Journal of community & applied social psychology, 16(6), 418–425.

[19] Steward W. T., Herek G. M., Ramakrishna J., Bharat S., Chandy S., Wrubel J., et al (2008). HIV-related stigma: adapting a theoretical framework for use in India. Social science & medicine, 67(8), 1225–1235. 18599171 10.1016/j.socscimed.2008.05.032 PMC2603621

[20] Earnshaw VA, Chaudoir SR. From conceptualizing to measuring HIV stigma: A review of HIV stigma mechanism measures. AIDS Behav 2009;13:1160–1177. 10.1007/s10461-009-9593-3 19636699 PMC4511707

[21] Herek G. M., & Capitanio J. P. (1998). Symbolic prejudice or fear of infection? A functional analysis of AIDS-related stigma among heterosexual adults. Basic and applied social psychology, 20(3), 230–241.

[22] Corrigan P. W., & Watson A. C. (2002). The paradox of self‐stigma and mental illness. Clinical Psychology: Science and Practice, 9(1), 35–53.

[23] Earnshaw V. A., Smith L. R., Chaudoir S. R., Amico K. R., & Copenhaver M. M. (2013). HIV stigma mechanisms and well-being among PLWH: a test of the HIV stigma framework. AIDS and Behavior, 17(5), 1785–1795. 10.1007/s10461-013-0437-9 23456594 PMC3664141

[24] Martinez J., Lemos D., & Hosek, and the Adolescent Medicine Trials Network, S. (2012). Stressors and sources of support: The perceptions and experiences of newly diagnosed Latino youth living with HIV. AIDS patient care and STDs, 26(5), 281–290. 10.1089/apc.2011.0317 22536931 PMC3335135

[25] Harper G. W., Lemos D., Hosek S. G., & Adolescent Medicine Trials Network for HIV/AIDS Interventions. (2014). Stigma reduction in adolescents and young adults newly diagnosed with HIV: Findings from the project ACCEPT intervention. AIDS patient care and STDs, 28(10), 543–554. 10.1089/apc.2013.0331 25216106 PMC4183905

[26] Ramos J. V., Mmbaga B. T., Turner E. L., Rugalabamu L. L., Luhanga S., Cunningham C. K., et al (2018). Modality of primary HIV disclosure and association with mental health, stigma, and antiretroviral therapy adherence in Tanzanian youth living with HIV. AIDS patient care and STDs, 32(1), 31–37. 10.1089/apc.2017.0196 29323556 PMC5756938

[27] Lall P., Lim S. H., Khairuddin N., & Kamarulzaman A. (2015). An urgent need for research on factors impacting adherence to and retention in care among HIV‐positive youth and adolescents from key populations. Journal of the International AIDS Society, 18, 19393 10.7448/IAS.18.2.19393 25724503 PMC4344535

[28] Lee S., Yamazaki M., Harris D. R., Harper G. W., & Ellen J. (2015). Social support and human immunodeficiency virus-status disclosure to friends and family: implications for human immunodeficiency virus-positive youth. Journal of Adolescent Health, 57(1), 73–80. 10.1016/j.jadohealth.2015.03.002 25940217 PMC4478132

[29] Rao D., Kekwaletswe T. C., Hosek S., Martinez J., & Rodriguez F. (2007). Stigma and social barriers to medication adherence with urban youth living with HIV. AIDS care, 19(1), 28–33. 10.1080/09540120600652303 17129855

[30] Wang C., & Burris M. A. (1997). Photovoice: Concept, methodology, and use for participatory needs assessment. Health education & behavior, 24(3), 369–387. 9158980 10.1177/109019819702400309

[31] Wang C. C. (1999). Photovoice: A participatory action research strategy applied to women's health. Journal of women's health, 8(2), 185–192. 10.1089/jwh.1999.8.185 10100132

[32] Teti M., Pichon L., Kabel A., Farnan R., & Binson D. (2013). Taking pictures to take control: Photovoice as a tool to facilitate empowerment among poor and racial/ethnic minority women with HIV. Journal of the Association of Nurses in AIDS Care, 24(6), 539–553. 10.1016/j.jana.2013.05.001 24064314 PMC3883445

[33] Booth T., & Booth W. (2003). In the frame: Photovoice and mothers with learning difficulties. Disability & Society, 18(4), 431–442.

[34] Ricoeur P. (1976). Interpretation theory: Discourse and the surplus of meaning. TCU press.

[35] Van Manen M. (1990). Researching lived experience. Human science for an action sensitive pedagogy. New York: State University of New York Press.

[36] Lindseth A., & Norberg A. (2004). A phenomenological hermeneutical method for researching lived experience. Scandinavian journal of caring sciences, 18(2), 145–153. 10.1111/j.1471-6712.2004.00258.x 15147477

[37] Laverty S. M. (2003). Hermeneutic phenomenology and phenomenology: A comparison of historical and methodological considerations. International journal of qualitative methods, 2(3), 21–35.

[38] Berger B. E., Ferrans C. E., & Lashley F. R. (2001). Measuring stigma in people with HIV: Psychometric assessment of the HIV stigma scale. Research in nursing & health, 24(6), 518–529. 11746080 10.1002/nur.10011

[39] Kalichman S. C. (2013). The harms of internalized AIDS stigma: a comment on Tsai et al. Annals of Behavioral Medicine, 46(3), 256–257. 10.1007/s12160-013-9529-z 23843080

[40] Crocker J. (1999). Social stigma and self-esteem: Situational construction of self-worth. Journal of experimental social psychology, 35(1), 89–107.

[41] Mutumba M., Bauermeister J. A., Musiime V., Byaruhanga J., Francis K., Snow R. C., et al (2015). Psychosocial challenges and strategies for coping with HIV among adolescents in Uganda: a qualitative study. AIDS patient care and STDs, 29(2), 86–94. 10.1089/apc.2014.0222 25607900

[42] Abubakar A., Van de Vijver F. J., Fischer R., Hassan A. S., Gona J. K., Dzombo J. T., et al (2016). ‘Everyone has a secret they keep close to their hearts’: challenges faced by adolescents living with HIV infection at the Kenyan coast. BMC public health, 16(1), 197. 26927422 10.1186/s12889-016-2854-y PMC4772469

[43] Bogart L. M., Cowgill B. O., Kennedy D., Ryan G., Murphy D. A., Elijah J., et al (2008). HIV-related stigma among people with HIV and their families: a qualitative analysis. AIDS and Behavior, 12(2), 244–254. 10.1007/s10461-007-9231-x 17458691

[44] Kelly M. J. (2000). Standing Education on its Head: Aspects of Schooling in a World with HIV/AIDS. Current issues in comparative education, 3(1), 28–38.

[45] Freeman M., & Nkomo N. (2006). Guardianship of orphans and vulnerable children. A survey of current and prospective South African caregivers. Aids care, 18(4), 302–310. 10.1080/09540120500359009 16809107

[46] Anderson K. L. (2010). Conflict, power, and violence in families. Journal of Marriage and Family, 72(3), 726–742.

[47] Crivello G., & Chuta N. (2012). Rethinking orphanhood and vulnerability in Ethiopia. Development in Practice, 22(4), 536–548.

[48] Stutterheim S. E., Pryor J. B., Bos A. E., Hoogendijk R., Muris P., & Schaalma H. P. (2009). HIV-related stigma and psychological distress: the harmful effects of specific stigma manifestations in various social settings. Aids, 23(17), 2353–2357. 10.1097/QAD.0b013e3283320dce 19741478

[49] Leary M. R., & Baumeister R. F. (2017). The need to belong: Desire for interpersonal attachments as a fundamental human motivation In Interpersonal development (pp. 57–89). Routledge. 7777651

[50] Busza J., Besana GV, Mapunda P., & Oliveras E. (2014). Meeting the needs of adolescents living with HIV through home based care: lessons learned from Tanzania. Children and youth services review, 45, 137–142.

[51] Mwai G. W., Mburu G., Torpey K., Frost P., Ford N., & Seeley J. (2013). Role and outcomes of community health workers in HIV care in sub‐Saharan Africa: a systematic review. Journal of the International AIDS Society, 16(1), 18586. 24029015 10.7448/IAS.16.1.18586 PMC3772323

[52] Alesina A., & Giuliano P. (2010). The power of the family. Journal of Economic growth, 15(2), 93–125.

[53] Marco, B. B., Jagero, N., & Abeka, S. O. (2011). Domestic violence in relation to children rights in Dar es Salaam, Tanzania.

[54] Helms C. B., Turan J. M., Atkins G., Kempf M. C., Clay O. J., Raper J. L., et al (2017). Interpersonal mechanisms contributing to the association between HIV-related internalized stigma and medication adherence. AIDS and Behavior, 21(1), 238–247. 10.1007/s10461-016-1320-2 26864692 PMC4980279

[55] Turan B., Smith W., Cohen M. H., Wilson T. E., Adimora A. A., Merenstein D., et al (2016). Mechanisms for the negative effects of internalized HIV-related stigma on antiretroviral therapy adherence in women: the mediating roles of social isolation and depression. Journal of acquired immune deficiency syndromes (1999), 72(2), 198 10.1097/QAI.0000000000000948 26885803 PMC4868649

[56] Vanable P. A., Carey M. P., Blair D. C., & Littlewood R. A. (2006). Impact of HIV-related stigma on health behaviors and psychological adjustment among HIV-positive men and women. AIDS and Behavior, 10(5), 473–482. 10.1007/s10461-006-9099-1 16604295 PMC2566551

[57] Drolet M., & Arcand I. (2013). Positive Development, Sense of Belonging, and Support of Peers among Early Adolescents: Perspectives of Different Actors. International Education Studies, 6(4), 29–38.

[58] Ambati B. K., Ambati J., & Rao A. M. (1997). Dynamics of knowledge and attitudes about AIDS among the educated in southern India. AIDS care, 9(3), 319–330. 10.1080/09540129750125118 9290837

[59] Brown L., Macintyre K., & Trujillo L. (2003). Interventions to reduce HIV/AIDS stigma: what have we learned?. AIDS education and prevention, 15(1), 49–69. 10.1521/aeap.15.1.49.23844 12627743

[60] Aguwa E. N., Arinze-Onyia S. U., Okwaraji F., & Modebe I. (2016). Assessment of Workplace Stigma and Discrimination among People Living with HIV/AIDS Attending Antiretroviral Clinics in Health Institutions in Enugu, Southeast Nigeria. West Indian Medical Journal, 65(1). 10.7727/wimj.2014.228 26645598

[61] Bruton, P. J., Rai, T., Day, S. E., Higgs, C., Rowlands, J., & Ward, H. (2017). P4. 18 Not so normalised–patient perspectives on hiv diagnosis and treatment decisions: results from a large qualitative study in london.

[62] Dlamini P. S., Wantland D., Makoae L. N., Chirwa M., Kohi T. W., Greeff M., et al (2009). HIV stigma and missed medications in HIV-positive people in five African countries. AIDS patient care and STDs, 23(5), 377–387. 10.1089/apc.2008.0164 19327098 PMC2716129

[63] Orban LA, Stein R., Koenig LJ, Conner LC, Rexhouse EL, Lewis JV et al (2010). Coping strategies of adolescents living with HIV: Disease-specific stressors and responses. AIDS Care, 22 (4), 420–430. 10.1080/09540120903193724 20146110

[64] Winchester M. S., McGrath J. W., Kaawa-Mafigiri D., Namutiibwa F., Ssendegye G., Nalwoga, et al (2013). Early HIV disclosure and nondisclosure among men and women on antiretroviral treatment in Uganda. AIDS care, 25(10), 1253–1258. 10.1080/09540121.2013.764386 23356654 PMC3657329

[65] Birungi H., Mugisha J. F., Obare F., & Nyombi J. K. (2009). Sexual behavior and desires among adolescents perinatally infected with human immunodeficiency virus in Uganda: implications for programming. Journal of Adolescent Health, 44(2), 184–187. 10.1016/j.jadohealth.2008.06.004 19167668

[66] Mukolo A., Blevins M., Victor B., Vaz L. M., Sidat M., & Vergara A. (2013). Correlates of social exclusion and negative labeling and devaluation of people living with HIV/AIDS in rural settings: evidence from a general household survey in Zambézia Province, Mozambique. PloS one, 8(10), e75744 10.1371/journal.pone.0075744 24146771 PMC3795715

[67] Gilbert L., & Walker L. (2010). ‘My biggest fear was that people would reject me once they knew my status…’: stigma as experienced by patients in an HIV/AIDS clinic in Johannesburg, South Africa. Health & social care in the community, 18(2), 139–146. 19708868 10.1111/j.1365-2524.2009.00881.x

[68] Carter-Black J. (2007). Teaching cultural competence: An innovative strategy grounded in the universality of storytelling as depicted in African and African American storytelling traditions. Journal of Social Work Education, 43(1), 31–50.

